# Studying the Effects of Two Various Methods of Composting on the Degradation Levels of Polycyclic Aromatic Hydrocarbons (PAHs) in Sewage Sludge

**DOI:** 10.1007/s11270-017-3481-7

**Published:** 2017-07-26

**Authors:** Joanna Poluszyńska, Elżbieta Jarosz-Krzemińska, Edeltrauda Helios-Rybicka

**Affiliations:** 1 Institute of Ceramic and Building Materials, Oświęcimska 21 St., 45-641 Opole, Poland; 2 AGH University of Science and Technology, 30 Mickiewicza Av., 30-059 Kraków, Poland

**Keywords:** Polycyclic aromatic hydrocarbons, PAHs, Sewage sludge, Vermicomposting, *Eisenia fetida*, Sawdust, Compost

## Abstract

The research comprised of studying the effect composting sewage sludge with sawdust and vermicomposting with earthworm *Eisenia fetida* has on the degradation of 16 polycyclic aromatic hydrocarbons (PAHs). Raw rural sewage sludge prior composting was more contaminated with PAHs than urban sewage sludge, in both cases exceeding EU cutoff limits of 6 mg/kg established for land application. Dibenzo[a,h]anthracene (DBahAnt), acenaphtylene (Acy) and indeno[1,2,3-c,d]pyrene (IPyr) were predominant in rural sewage sludge, whilst the urban sewage sludge contained the highest concentrations of benzo[b]fluoranthene (BbFl), benzo[k]fluoranthene (BkFl) and indeno[1,2,3-c,d]pyrene (IPyr). Thirty days of composting with sawdust has caused a significant reduction of 16 PAHs on average from 26.07 to 4.01 mg/kg (84.6%). During vermicomposting, total PAH concentration decreased on average from 15.5 to 2.37 mg/kg (84.7%). Vermicomposting caused full degradation of hydrocarbons containing 2 and 6 rings and significant reduction of PAHs with 3 aromatic rings (94.4%) as well as with 5 aromatic rings (83.2%). The lowest rate of degradation (64.4%) was observed for hydrocarbons with 4 aromatic rings such as fluoranthene, benzo(a)anthracene, chrysene and pyrene. On the other hand, the highest level of degradation was determined for PAHs with 2 rings (100%), 3 rings (88%) and 6 aromatic rings in the molecule (86.9%) after composting with sawdust. Acenaphthene and pyrene were found to be the most resistant to biodegradation during both composting methods.

## Introduction

Sewage sludge is an organic waste material which, when properly treated, may become an inexpensive source of nutrients used e.g. as a substitute of agricultural fertilizers. Since this specific waste is a by-product of wastewater treatment, its composition varies significantly depending on the origin of the incoming wastewater to the plant. Raw sewage sludge, besides containing valuable elements such as nitrogen, phosphorus, calcium, magnesium or sulfur is also rich with potentially dangerous contaminants such as heavy metals, pathogenic organisms or undesired organic substances of industrial origin, which mostly exclude this type of waste from landfilling or direct agricultural and land use. In European countries thermal incineration of sewage sludge is the most preferable utilization solution applicable to this type of waste (Tyagi and Lo 2013). However, since its costs are significant because of the necessity of drying the sludge prior to incineration, thermal treatment is employed mainly in large urban wastewater treatment plants, whilst rarely applicable in small plants. The most common methods employed in stabilization and hygienization of raw sewage sludge is liming, composting or passive drying. Composting of the sewage sludge is in fact decomposition (mineralization) and humification of the waste biomass. This process undergoes completion with the use of microorganisms and can be carried out in an open system heaps or in special bioreactors. Treatment of sewage sludge is mandatory in all treatment plants. It decreases the volume and mass of the sludge and it leads to the production of quality organic fertilizer.

Polycyclic aromatic hydrocarbons (PAHs) are the most common group of organic contaminants present in elevated concentrations in sewage sludge. Due to their hydrophobic nature they are removed during the wastewater biological treatment from the water phase by adsorption onto suspended solids and hence they are accumulated in the sludge (Amir et al. 2005). The group of polycyclic aromatic hydrocarbons contains approximately 200 compounds; however, sixteen of them are identified by the United States Environmental Protection Agency as “priority pollutants” and seven namely benzo(a)anthracene, chrysene, benzo(a)pyrene, benzo(b)fluoranthene, benzo(k)fluoranthene, dibenz(a,h)anthracene, and indeno(1,2,3-cd) pyrene are described as probably carcinogenic. Table 1 contains the full list of the 16 PAH compounds targeted by the USEPA agency. As depicted in Table 1, in general, the carcinogenic PAHs have a higher molecular weight, number of aromatic rings and lower vapor pressure, as well as solubility constants when compared to the non-carcinogenic PAHs (Bojes and Pope 2007).

**Table 1:**
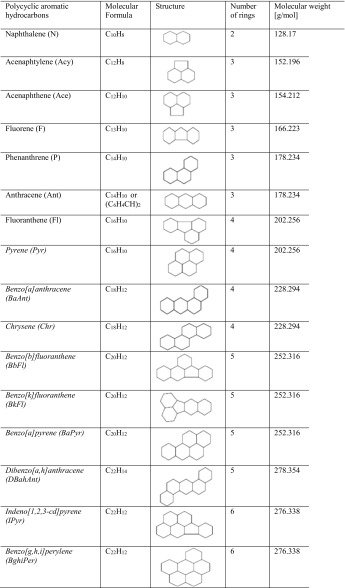
List of 16 polycyclic aromatic hydrocarbons

In italics- PAHs classified as probable human carcinogens according to US EPA

PAH content in sewage sludge can vary significantly, ranging from <6 mg/kg d.m. up to a few hundred milligramme/kilogramme d.m. depending on the country and the origin of incoming wastewater to the plant. In Poland, for instance, Oleszczuk (2007, 2009) reports that in raw sewage sludge, the sum of 16 PAHs ranges from 2.83 to 10.35 mg/kg d.m. Moreover, Paulsrud et al. (1997) reveals that in Denmark, PAH concentration in sewage sludge varies from 1.0 up to even 30 mg/kg d.m. On the other side of the globe, in China, researchers Chang et al. (2002), Chang et al. (2003), Feng et al. (2008), Liu et al. (2013), Cai et al. (2007) and Li et al. (2008) report that the sum of 16 PAHs in the tested raw sewage sludge can be as little as 0.5 mg/kg d.m. or as high as 100.74 mg/kg d.m.

Poland so far has not yet defined the maximum permissible content of PAHs in sewage sludge used as fertilizer. It has introduced only (as other EU countries) limits for heavy metal content in sewage sludge applied to soil. However, the third amendment to the Directive 1986/278/EEC (Working Document on Sludge 3^rd^ Draft 2000) introduces a cutoff value of 6 mg/kg d.m. for the total concentration of 11 PAHs (including acenaphthene, phenanthrene, fluorene, fluoranthene, pyrene, benzo(b)fluoranthene, benzo(j)fluoranthene, benzo(k)fluoranthene, benzo(a)pyrene, benzo(g,h,i)perylene, indeno(1,2,3-cd pyrene)) in sewage sludge intended for agricultural or land application. According to data from 2001 and 2008, only four European Union countries have introduced limits for PAHs in sewage sludge. These were Austria (Carinthia) with a limit of 6 mg/kg d.m., Denmark and Sweden with a limit of 3 mg/kg d.m. and France with a limit of 4 mg fluoranthene/kg d.m., 2.5 mg of benzo (b) fluoranthene/kg d.m. and 1.5 benzo (a) pyrene/kg d.m. in the sludge. These countries have also defined the frequency of PAH analysis in sewage sludge intended for use as soil fertilizers.

Due to the fact that the use of composted sewage sludge as soil fertilizer is a common practice, knowledge on how the stabilization process affects the reduction of contaminants is considered crucial. In recent years, numerous research addressed the issue of heavy metals immobilization in composted sewage sludge (Singh and Kalamdhad 2012, 2013; Branzini and Zubillaga 2012; Gul et al. 2015). However, substantially less research was conducted on the impact that the composting process has on PAH degradation.

The aim of the research was to determine the effect of two methods of sewage sludge composting, using sawdust and vermicomposting with *Eisenia fetida* have on the level of PAH degradation.

## Materials and Methods

### Materials

Two types of sewage sludge collected from urban and rural municipal sewage plants, located in Opole Voivodeship (Southern Poland), were used in the study. Each sewage sludge was composted using a different method. Rural sewage treatment plant was using heap composting method. The raw sewage sludge prior to composting was concentrated in a gravity sludge concentrator and dehydrated by a belt press with the addition of polyelectrolyte. Dehydrated sludge was mixed with sawdust and placed on compost heaps, without shifting and additional aeration. Urban sewage treatment plant applied a vermicomposting method using earthworm *E. fetida.* This process of composting was carried out in sections with a width of 4 m and length of 100 m. Thickness of the composted material ranged from 60 to 80 cm.

**Fig. 1:**
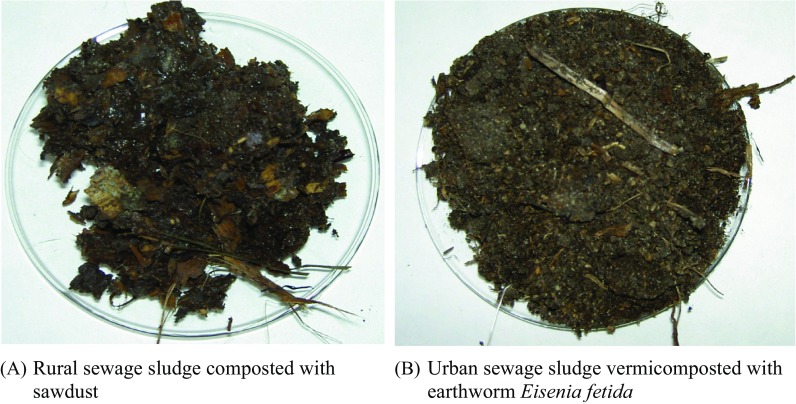
Samples of sewage sludge composted using sawdust (**a**) and vermicomposting with earthworm *E. fetida* (**b**)

Both compost prisms with sawdust and vermicompost sections were located on site of the wastewater treatment plants. Samples of raw sewage sludge from both rural and urban wastewater plants were collected monthly for 6 months, starting from May to October 2010. Resultant compost samples were collected and examined after 30 days of composting Fig. 1.

### PAH Extraction

A protocol for the determination of PAH concentration in raw sewage sludge and the final compost was developed in the Laboratory of Innovative Materials and Environmental Monitoring ICiMB in Opole, Poland. Samples were dried at 20 °C then ground and sieved through a Ø 0.7–1.0 mm sieve. Five grammes of the prepared samples was then extracted with a mixture of hexane: acetone 4:1 *v*/*v* (10 cycles) in FexIka apparatus, concentrated under nitrogen to the volume of 0.5 cm^3^ and then purified on a solid-phase extraction columns (SPE) filled with the layer of sodium sulfate and silica gel.

### GC Analysis

The purified and concentrated extracts were analyzed using gas chromatograph DANI GC-1000 with flame ionization detector (FID). Capillary column DB-5 (length 30 m × 0.25 mm × 0.25 um) was used in the analysis. Detailed experimental conditions for the GC analysis are presented in Table 2.

**Table 2:** Experimental conditions for PAH analysis using gas chromatography method

Parameter	Condition
GC oven temperature program	100 °C for 3 min, temperature increase 5 °C/min to 300 °C, 300 °C—maintained for 10 min
Injector temperature	300 °C
Detector temperature	300 °C
The flow through the column carrier gas (helium)	1.0 cm^3^/min

A standard solution of 500 μg/cm^3^ concentration was prepared according to EPA requirements as a mixture of 16 PAHs: naphthalene (N), acenaphthylene (Acy), acenaphthene (Ace), fluorene (F), phenanthrene (P), anthracene (Ant), fluoranthene (Fl), pyrene (Pyr), benzo[a]anthracene (BaAnt), chrysene (Chr), benzo[b]fluoranthene (BbFl), benzo[k]fluoranthene (BkFl), benzo[a]pyrene (BaPyr), dibenzo[a,h]anthracene (DBahAnt), indeno[1,2,3-c, d]pyrene (Ipyr) and benzo [g,h,i]perylene (BghiPer). The individual compounds were then identified based on the comparison of the retention time between samples and the standard solution of 16 PAHs. GC analytical detection limits for all 16 PAH concentration was equal to 0.04 mg/kg d.m.

### Data Quality

Certified reference material was used in order to investigate PAH recovery. Quantitative recovery parameters for all determined PAHs ranged between 81 and 120%, which is permissible in chromatography analysis.

Qualitative evaluation was performed by comparing of the relative retention times for each of the 16 PAHs from calibration standard with the relative retention times of compounds in analyzed samples. Additionally, the results were confirmed by analyzing mass spectra characteristic for the individual PAHs using Shimadzu gas chromatography equipped with a mass spectrometry detector GC-MS. The very same temperature program was employed along with the same calibration standard and chromatography column as was used during the GC-FID analysis.

## Results and Discussion

### Concentrations of PAHs in Raw Sewage Sludge Before Composting

Both types of uncomposted, raw sewage sludge from rural and urban wastewater treatment plants have revealed high concentrations of all 16 PAHs, significantly exceeding the EU cutoff limit of 6 mg/kg d.m.. Benzo[b]fluoranthene and benzo[k]fluoranthene were the most commonly found PAHs in all tested samples, whereas carcinogenic benzo[a]pyrene, considered a PAH indicator, rarely occurred and in most cases was found to be below the detection limit of the analytical method (0.04 mg/kg d.m.). Both types of sewage sludge differ in PAH composition and concentrations of the individual compounds. Raw sewage sludge from the rural wastewater treatment plant was slightly more contaminated with PAHs than the sewage sludge derived from urban wastewater treatment plant, because of an extremely high content of acenaphtylene and dibenzo[a,h]anthracene. The concentrations of the remaining PAHs in both types of raw sewage sludge were comparable and the details are summarized in Table 3. In rural sewage sludge, the highest concentrations were determined for hydrocarbons with higher molecular weight such as indeno[1,2,3-c,d]pyrene (concentration from 3.79 to 5.60 mg/kg) or dibenzo[a,h]anthracene (4.69 up to 10.86 mg/kg), but also for acenaphthylene (2.27–7.41 mg/kg). Urban sewage sludge was however contaminated predominantly with benzo(b)fluoranthene (2.25 to 7.17 mg/kg) and benzo(k)fluoranthene (1.71 to 5.06 mg/kg), and also most often commonly occuring in all samples. Even higher concentrations (5.29 in sample 7 – 2010 and up to 9.77 in sample 2 – 2010) were found for indeno(1,2,3-cd) pyrene; however, this occurred only in four sewage sludge samples. Concentrations of the remaining PAHs did not exceed the concentration of 1 mg/kg except for the dibenzo[a,h]anthracene and benzo(g,h,i)perylene, which were not detected in any of the examined sludge. Carcinogenic benzo[a] pyrene was detected only in one sample (1-2010) and its concentration was equal to 0.69 mg/kg. The sum of 16 PAHs in rural sewage sludge varied, ranging between 19.48 and 30.53 and in sewage sludge from urban wastewater plant from 10.06 to 25.29 mg/kg d.m. (Table 4).

**Table 3:** Concentrations of individual PAHs in raw rural and urban sewage sludge

Sample	Concentration [mg/kg]															
	N	Acy	Ace	F	P	Ant	Fl	Pyr	BaAnt	Chr	BbFl	BkFl	BaPyr	IPyr	DBahAnt	BghiPer
Raw rural sewage sludge																
1 – 2010	0.61	7.41	0.62	0.35	1.02	0.72	–	0.64	0.23	0.43	1.06	2.02	1.30	3.79	4.69	5.65
2 – 2010	1.18	2.27	0.23	0.23	1.13	1.07	0.28	1.12	0.39	0.75	0.74	0.79	–	4.98	10.86	–
3 – 2010	1.74	5.17	0.35	0.10	1.96	0.95	0.27	0.93	0.68	0.69	0.96	0.74	–	5.60	9.53	0.79
4 – 2010	1.74	4.19	0.62	0.25	1.34	2.65	–	–	0.39	0.55	0.34	0.35	–	4.41	8.64	–
5 – 2010	1.25	3.28	1.11	0.17	1.51	1.35	–	–	0.40	0.49	0.55	0.93	–	3.21	5.23	–
6 – 2010	0.98	4.15	0.85	0.23	1.43	1.97	0.38	0.54	0.52	0.24	0.72	0.71	–	4.12	7.18	0.44
Raw urban sewage sludge																
1 – 2010	0.69	0.92	0.09	–	1.71	0.34	–	0.83	0.39	0.46	2.25	1.71	0.69	–	–	–
2 – 2010	–	–	0.39	0.10	0.53	0.06	0.50	0.55	–	0.61	4.50	2.95	–	9.77	–	–
3 – 2010	0.34	0.17	0.11	–	0.81	0.72	0.54	0.82	0.37	0.54	7.17	5.36	–	8.35	–	–
4 – 2010	0.44	0.67	0.20	–	1.13	0.46	–	0.72	–	0.50	5.61	4.00	–	–	–	–
5 – 2010	–	0.15	0.30	–	0.98	0.45	–	0.67	–	0.52	3.54	4.17	–	–	–	–
6 – 2010	0.62	0.44	0.14	–	0.59	0.27	0.40	0.52	0.40	0.48	4.28	5.06	–	–	–	–

– not detected

**Table 4:** Sixteen PAH concentrations according to EPA and 11 PAH concentrations according to WD2000 in raw rural and urban sewage sludge

Concentration [mg/kg d.m.]		
PAHs	Raw rural sewage sludge	Raw urban sewage sludge
Naphthalene (N)	0.61–1.74	<0.04–0.69
Acenaphtylene (Acy)	2.27–7.41	<0.04–0.92
Acenaphtene (Ace)	0.23–0.62	0.09–0.39
Fluorene (F)	0.10–0.35	<0.04–0.10
Phenanthrene (P)	1.02–1.96	0.53–1.71
Anthracene (Ant)	0.72–2.65	0.06–0.72
Fluoranthene (Fl)	<0.04–0.28	<0.04–0.54
Pyrene (Pyr)	<0.04–1.12	0.55–0.83
Benzo[a]anthracene (BaAnt)	0.23–0.68	<0.04–0.39
Chrysene (Chr)	0.43–0.75	0.46–0.61
Benzo[b]fluoranthene (BbFl)	0.34–1.06	2.25–7.17
Benzo[k]fluoranthene (BkFl)	0.35–2.02	1.71–5.36
Benzo[a]pyrene (BaPyr)	<0.04–1.30	<0.04–0.69
Dibenzo[a,h]anthracene (DBahAnt)	4.69–10.86	<0.04
Indeno[1,2,3-c,d]pyrene (IPyr)	3.79–5.60	<0.04–9.77
Benzo[g,h,i]perylene (BghiPer)	<0.04–5.65	<0.04
Ʃ 16 PAHs acc. EPA	19.48–30.54	10.06–25.29
Ʃ 11 PAHs acc WD2000	7.30–16.44	7.27–23.16

### Concentration of PAHs in the Final Resultant Compost Samples

Composting of sewage sludge with sawdust has led to a significant reduction of all examined PAHs, especially naphthalene (N), acenaphtylene (Acy), fluorene (F), fluoranthene (Fl), indeno[1,2,3-c,d]pyrene (IPyr), dibenzo[a,h]anthracene (DBahAnt) and benzo[g,h,i]perylene (BghiPer). Dibenzo[a,h]anthracene (DBahAnt) was the most biodegradable of all the PAHs, except for one compost sample in which the concentration of this compound remained as high as 5.04 mg/kg. Concentrations of the individual PAHs in the resultant compost with sawdust are presented in Table 5. The sum of 11 PAHs in the final compost ranged from 1.57 to 4.94 mg/kg d.m. and thus met the requirements of the proposed cutoff limit of 6 mg/kg d.m.

**Table 5:** Concentrations of individual PAHs in the final composts

Sample	Concentration [mg/kg]															
	N	Acy	Ace	F	P	Ant	Fl	Pyr	BaAnt	Chr	BbFl	BkFl	BaPyr	IPyr	DBahAnt	BghiPer
Compost with sawdust																
1–2010	–	0.21	0.45	0.30	0.28	0.58	–	0.30	–	–	0.48	0.29	0.52	2.32	–	–
2–2010	–	0.04	0.13	–	0.17	0.28	–	–	–	–	0.61	0.67	–	–	–	–
3–2010	–	–	0.39	–	0.53	0.06	–	0.55	–	0.61	0.75	0.40	–	–	–	–
4–2010	–	–	0.18	–	0.83	0.34	–	–	0.25	0.44	0.80	0.10	–	2.07	5.04	–
5–2010	–	–	0.04	0.04	0.22	–	–	0.09	–	–	0.20	0.32	–	–	–	–
6–2010	–	0.11	0.10	–	0.50	0.22	–	0.05	–	–	0.35	0.46	–	0.33	0.06	–
Vermicompost																
1–2010	–	–	0.09	–	0.16	0.14	–	0.63	0.28	0.34	0.35	0.41	–	–	–	–
2–2010	–	–	0.20	0.10	–	–	–	0.18	–	–	0.55	0.31	–	–	–	–
3–2010	–	–	–	–	–	–	0.48	0.59	0.33	0.52	2.14	1.14	–	–	–	–
4–2010	–	–	–	–	–	–	–	0.30	–	–	1.04	0.79	–	–	–	–
5–2010	–	–	–	–	–	0.10	–	0.31	–	–	0.24	0.50	–	–	–	–
6–2010	–	–	0.04	–	–	–	0.05	0.20	0.25	0.06	1.30	0.14	–	–	–	–

– not detected

Slightly more efficient biodegradation of PAHs was reported during composting of sewage sludge using *E. fetida* vermicomposting method. In the final compost, complete degradation was reported for naphthalene (N), acenaphtylene (Acy), benzo[a]pyrene (BaPyr) and indeno[1,2,3-c,d]pyrene (IPyr). The concentration of the remaining PAHs ranged from 0.04 to 2.14 mg/kg d.m. Indeno[1,2,3-c,d]pyrene (IPyr) was the best degrading of all examined PAHs during vermicomposting. The sum of 11 PAHs in vermicompost varied from 1.34 to 4.35 [mg/kg d.m.] and is also below the proposed cutoff limit of 6 mg/kg d.m. (Table 6 **)**. On average, the total concentration of PAHs was reduced by 84.7% of their initial level. This appears to correspond with the results of other authors, determined in laboratory scale experiments on vermicomposting, which report comparable levels of PAH degradations (Contreras-Ramos et al. 2008; Rorat et al. 2017).

**Table 6:** Sixteen and 11 PAH concentrations in final resultant compost samples

Concentration [mg/kg d.m.]		
PAHs	Compost with sawdust	Vermicompost
Naphthalene (N)	<0.04	<0.04
Acenaphtylene (Acy)	<0.04–0.21	<0.04
Acenaphtene (Ace)	0.13–0.45	<0.04–0.20
Fluorene (F)	<0.04–0.30	<0.04–0.10
Phenanthrene (P)	0.17–0.83	<0.04–0.16
Anthracene (Ant)	0.06–0.58	<0.04–0.14
Fluoranthene (Fl)	<0.04	<0.04–0.48
Pyrene (Pyr)	<0.04–0.55	0.18–0.63
Benzo[a]anthracene (BaAnt)	<0.04–0.25	<0.04–0.33
Chrysene (Chr)	<0.04–0.61	<0.04–0.52
Benzo[b]fluoranthene (BbFl)	0.48–0.80	0.35–2.14
Benzo[k]fluoranthene (BkFl)	0.10–0.67	0.31–1.14
Benzo[a]pyrene (BaPyr)	<0.04–0.52	<0.04
Dibenzo[a,h]anthracene (DBahAnt)	<0.04–5.04	<0.04
Indeno[1,2,3-c,d]pyrene (IPyr)	<0.04–2.32	<0.04
Benzo[g,h,i]perylene (BghiPer)	<0.04	<0.04
Ʃ 16 PAHs acc. EPA	1.89–10.04	1.34–5.21
Ʃ 11 PAHs acc. WD2000	1.57–4.94	1.34–4.35

### Degradation Level of PAHs in Sewage Sludge Composted with Sawdust

All 16 PAHs were partially or fully degraded during composting of sewage sludge with sawdust. Average reduction of the individual compounds ranged from 74.2% for PAHs with 4 rings in the molecule, to 100% reduction with respect to 2-ring naphthalene. Table 7 depicts the level of degradation for individual PAHs in the final compost with sawdust. The obtained results are in line with the 2007 Oleszczuk study, in which it was reported that the hydrocarbons with the smallest number of aromatic rings in the molecule are the most mobile ones and consequently most available to the action of bacteria, fungi as well as enzymes from other organisms living in the compost. These hydrocarbons, due to weaker hydrophobic properties than those with more rings in the molecule (5–6 rings), are less associated with solid particles which in turn causes them to be more bioavailable (Oleszczuk 2007).

**Table 7:** PAH reduction [%] in final compost samples

Reduction percentage [%]					
	Numbers of rings				
	2	3	4	5	6
Compost with sawdust					
1 – 2010	100.0	82.0	76.9	85.8	75.4
2 – 2010	100.0	87.4	100.0	89.7	100.0
3 – 2010	100.0	88.5	54.9	89.8	100.0
4 – 2010	100.0	85.1	26.6	36.3	53.1
5 – 2010	100.0	96.0	89.9	92.3	100.0
6 – 2010	100.0	89.2	97.0	89.9	92.8
mean	100.0	88.0	74.2	80.6	86.9
Vermicompost					
1 – 2010	–	72.2	89.2	88.5	100.0
2 – 2010	100.0	100.0	15.4	73.8	100.0
3 – 2010	100.0	100.0	75.4	81.0	–
4 – 2010	100.0	97.2	68.9	84.6	–
5 – 2010	100.0	100.0	68.1	86.1	100.0
6 – 2010	100.0	96.8	69.2	85.5	100.0
mean	100.0	94.4	64.4	83.2	100.0

– not detected in raw sewage sludge

Except for one sample of compost, all sewage sludge composted with sawdust has met the recommended value of 6 mg/kg d.m. intended for agricultural use of sewage sludge, according to WD 2000.

### Degradation Level of PAHs in Sewage Sludge Composted with *E. fetida*

Vermicomposting which resulted in full degradation of hydrocarbons containing 2 and 6 rings, a significant reduction of PAHs with 3 aromatic rings (on average 94.4%) as well as those containing 5 rings (83.2%), confirms the presence of highly favorable conditions created by the action of earthworms on the biodegradation process. The lowest rate of degradation was observed for hydrocarbons with 4 rings (64.4%), such as fluoranthene, benzo(a)anthracene, chrysene and pyrene (Table 7).

All samples of vermicompost have met EU cutoff limits of 6 mg/kg d.m. for Σ16 and Σ11 of PAHs, fulfilling the requirements established by WD 2000 for land application. The obtained results confirm the findings of other authors (Siebielska and Sidełko 2009; Lazzari et al. 2000; Amir et al. 2005; Hua et al. 2008, Hamdi et al. 2006; Cai et al. 2007; Oleszczuk 2009) claiming that composting of sewage sludge or co-composting contaminated soil with sewage sludge can significantly reduce PAH content in this material. Specifically, degradation levels of 31.2 up to 90% as compared to the initial PAH content could be observed.

## Conclusions

Raw sewage sludge from the rural wastewater treatment plant was slightly more contaminated with PAHs than the sewage sludge derived from the urban wastewater treatment plant. This could be attributed to a high concentration of acenaphtylene and dibenzo[a,h]anthracene. Both composting methods i.e. vermicomposting using *E. fetida* and composting with sawdust were however very effective in biodegradation of Σ16 and Σ11 PAHs in all sewage sludge. The level of degradation was different for PAHs with 2 and 3 rings from that of 4 and more rings. After composting with sawdust or vermicomposting, the greatest reduction was found for PAHs with 2, 3, and 6 aromatic rings per molecule. Vermicomposting resulted in full degradation of hydrocarbons containing 2 and 6 rings as well as 94.4% reduction of PAHs with 3 aromatic rings in molecule and an 83.2% reduction of 5 rings PAHs. The lowest level of degradation (64.4%) during vermicomposting was determined for hydrocarbons with 4 aromatic rings. During composting of sewage sludge with sawdust, the highest removal rates were determined to be for PAHs with 2 aromatic rings in the molecule (100%), 3 rings (88%) and 6 aromatic rings (86.9%). In both composting methods, acenaphthene and pyrene were found to be the most resistant to biodegradation.

A very low concentration of benzo(a)pyrene in the final compost with sawdust (0.52 mg/kg d.m, 60% degradation rate) or complete lack of it in vermicompost (100% degradation) indicates that both types of derived compost do not cause any threat to soil environment with regards to PAH contamination. In order to minimize the risk of soil contamination with persistent organic pollutants (POPs), it is recommended that all EU countries introduce limit values for PAH concentrations in sewage sludge intended for agricultural use. Since PAH compounds exhibit different toxicological risk, threshold values mostly be established for benzo(a)pyrene, because it is the most carcinogenic of all PAHs. Ultimately, limits on PAH concentrations should also be introduced to all carcinogenic PAH compounds and to other persistent organic pollutants.
